# Heed The Warning: A Case Report on Capsular Warning Syndrome

**DOI:** 10.5811/cpcem.47259

**Published:** 2025-11-16

**Authors:** Targol Tarahomi, Sean Serio, Alexander John Scumpia

**Affiliations:** * HCA Florida Aventura Hospital, Department of Emergency Medicine, Aventura, Florida; † Baptist Health Baptist Hospital, Department of Emergency Medicine, Miami, Florida

**Keywords:** capsular warning syndrome, lacunar stroke, case report

## Abstract

**Introduction:**

Evaluating patients with acute neurologic deficits is a regular occurrence in the emergency department (ED), but some presentations warrant increased concern. This case highlights the importance of repeat evaluations and how resolution of symptoms does not rule out a more ominous underlying pathology.

**Case Report:**

A 59-year-old male with a past medical history of coronary artery disease and Human immunodeficiency viruses (HIV) presented to a Level II trauma and comprehensive stroke center for left-sided facial droop and left-sided hemiparesis. Computed tomography of the brain including angiography and perfusion was performed revealing no hemorrhage or large vessel occlusion. Given his National Institutes of Health Stroke Scale score of 11, he received alteplase and subsequently experienced several episodes of resolution and recurrence of his symptoms while in the ED. Magnetic resonance imaging revealed an acute ischemic infarct in the right basal ganglia and insular region, which along with his clinical presentation was consistent with capsular warning syndrome.

**Conclusion:**

Capsular warning syndrome is a rare clinical entity with an incidence ranging from 1.5–5% in stroke patients. Its recognition is crucial when making decisions concerning management, as resolution of symptoms should still garner a high level of attention given that the the increased risk of stroke with permanent neurological disability is highest within the first 48 hours. The role of thrombolysis continues to be an area of focus as its benefit has not yet been determined but continues to be the mainstay therapy in the correct clinical setting. This is especially true in the cases of recurrent episodes post thrombolysis, which does not preclude the diagnosis of capsular warning syndrome but should heighten the need for acute management of these patients and close monitoring. This case illustrates its unique presentation and the need for increased recognition and understanding within the field of emergency medicine.

## INTRODUCTION

Capsular warning syndrome is a rare clinical entity characterized by three or more recurrent episodes of motor and sensory deficits occurring within a 24-hour period, with complete resolution between episodes.^1^ This syndrome represents a distinct subtype of lacunar strokes, predominantly involving the basal ganglia, subcortical white matter, and pons, and is notable for the absence of cortical symptoms such as visual field deficits, neglect, and agnosia.

The pathophysiology of capsular warning syndrome is attributed to compromised blood flow in small, branching lacunar vessels, which typically results in unilateral motor or sensory deficits. The clinical significance of this syndrome cannot be overstated, as approximately one-third of transient ischemic attacks, of which lacunar infarcts constitute a part, are associated with subsequent cerebral infarction.^2^ The incidence rate of capsular warning syndrome ranges from 1.5–5%, highlighting the critical need for prompt recognition and management to mitigate the risk of progression to more severe cerebrovascular events. Early intervention is essential for improving patient outcomes and preventing the potential transition from transient ischemic episodes to definitive strokes.

## CASE REPORT

We present the case of a 59-year-old male with a past medical history significant for coronary artery disease managed with seven cardiac stents and a diagnosis of HIV, who presented to the ED as a stroke alert due to left-sided facial droop and hemiparesis, which began about two hours prior to arrival to the ED. Upon evaluation, the patient’s blood pressure was 155/80 millimeters of mercury, heart rate 63 beats per minute, and respiratory rate 20 respirations per minute, with an oxygen saturation of 100% on room air. The patient’s National Institutes of Health Stroke Scale (NIHSS) score. was 11, indicating partial facial paralysis, no effort against gravity in the left upper and lower extremities, and mild-to-moderate dysarthria.

Because the patient was within the therapeutic window for administration of alteplase—a tissue plasminogen activator—a collaborative decision was made with neurology consult and the patient to proceed with thrombolytic therapy, which was initiated 22 minutes after the patient’s arrival. Following treatment, he exhibited rapid symptom resolution, achieving a NIHSS score of zero within 10–15 minutes. However, approximately 15 minutes after initial resolution he subsequently experienced four episodes of recurrent stroke symptoms, each lasting between 3–10 minutes, with complete resolution between episodes. During these episodes, his symptoms were identical to the initial presentation, and all four episodes were within a span of 48 minutes.

Neuroimaging, including computed tomography (CT) angiography of the head and neck and CT perfusion, revealed benign oligemia in the vertebral-basilar posterior circulation without evidence of penumbral regions or infarct core. The patient was then admitted to the intensive care unit for close monitoring. A magnetic resonance imaging study performed the following day identified an acute ischemic infarct in the right basal ganglia and insular region (Image). A follow-up CT conducted 24 hours later demonstrated hypodensity in the right putamen consistent with a subacute infarct.

Throughout his hospitalization, the patient underwent a bilateral extremity ultrasound, which was negative for deep vein thrombosis, and a transesophageal echocardiogram that ruled out the presence of thrombus in the left atrial appendage and aortic atheroma. Optimization of his medication regimen included the initiation of dual antiplatelet therapy with aspirin and clopidogrel for a duration of 21 days; the need for close monitoring was discussed with the patient. He remained stable without neurological episodes for six days and was subsequently discharged to a rehabilitation facility for continued recovery and management. At time of discharge, the patient’s modified Rankin Score for neurological disability was zero.

*CPC-EM Capsule*

What do we already know about this clinical entity?

*Capsular warning syndrome represents a subtype of lacunar infarcts that involves episodic motor and sensory deficits within a 24-hour period.*

What makes this presentation of disease reportable?

*This description of episodes of capsular warning syndrome in the emergency department adds to the literature about this rare clinical entity.*

What is the major learning point?

*The transient nature of this syndrome can lead to incorrect recognition, thereby leading to improper management and disposition.*

How might this improve emergency medicine practice?

*Awareness of capsular warning syndrome could prevent the potential transition from transient ischemic episodes to definitive strokes*.

## DISCUSSION

Lacunar infarcts result from the involvement of small penetrating lenticulostriate arteries that are affected by hypertensive arteriolar sclerosis.^4^ These arteries are branches of the middle cerebral artery and supply blood to critical regions, including the basal ganglia—comprising the striatum, nucleus accumbens, and globus pallidus—as well as the subcortical white matter, including the internal capsule and corona radiata, and the pons. These areas play integral roles in voluntary movement and feedback regulation to the cortex via the thalamus, specifically through its anterior, posterior, and genu limbs. Lesions in these regions can lead to abnormalities in the corticobulbar tract, corticospinal tract, and posterolateral thalamus.

Capsular warning syndrome represents a specific subset of lacunar infarcts characterized by three or more episodes of transient motor or sensory deficits affecting the face and extremities. Pure motor deficits are typically associated with lesions in the internal capsule, whereas pure sensory deficits correspond to lesions in the thalamus. Identifiable risk factors for this syndrome include hypertension, diabetes mellitus, dyslipidemia, and smoking.^3^ The incidence of capsular warning syndrome ranges from 1–5% of all transient ischemic attacks, highlighting a significant risk for subsequent stroke development.^2^ Notably, the risk of stroke associated with permanent neurological disability is highest within the first 48 hours, with up to a 60% likelihood of stroke occurring within the first week following the initial episode.^3^

Management strategies remain challenging to delineate. Current therapeutic approaches primarily involve tissue plasminogen activator and dual antiplatelet therapy, although studies are inconclusive as to the therapeutic efficacy of these modalities.^5^ The range of benefit specifically for thrombolytics ranges from complete resolution of symptoms and cessation of any further stuttering episodes to no benefit at all. 5 The need for further research into optimal management strategies is evident, given the high associated risk of impending cerebrovascular events.

## CONCLUSION

Capsular warning syndrome serves as a critical clinical marker of impending lacunar infarcts, highlighting the need for heightened awareness among emergency clinicians. Recognizing the transient nature of the neurological deficits, along with the significant risk factors such as hypertension, diabetes, dyslipidemia, and smoking, is essential for early intervention and management. The association between capsular warning syndrome and an elevated risk of subsequent stroke underscores the importance of timely diagnostic evaluation and therapeutic decision-making. Although current treatment strategies primarily involve tissue plasminogen activator and dual antiplatelet therapy, further research is necessary to optimize management and improve patient outcomes. Ultimately, early recognition and management of capsular warning syndrome are pivotal to preventing progression to disabling cerebrovascular events and improving long-term outcomes.

## Figures and Tables

**Image:**
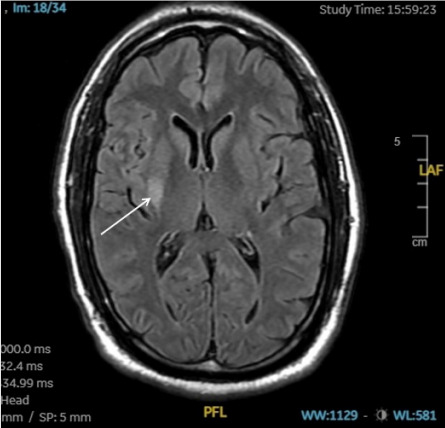
T2 FLAIR of magnetic resonance imaging brain revealing an acute ischemic infarct in the right basal ganglia and insular region (white arrow). *FLAIR*, fluid-attenuated inversion recovery.
